# Activity‐restoring mutations in the histamine H_3_ receptor increase constitutive activity and reduce structural stability

**DOI:** 10.1002/pro.70408

**Published:** 2025-12-22

**Authors:** Ami Nakajima, Hiroto Kaneko, Kosuke Oyama, Misumi Kuchiji, Ayane Itakura, Chiaki Arai, Mitsunori Shiroishi

**Affiliations:** ^1^ Department of Biological Science and Technology Tokyo University of Science Tokyo Japan

**Keywords:** activity‐restoring mutation, constitutive activity, histamine H_3_ receptor, receptor stability, *Saccharomyces cerevisia*e

## Abstract

The histamine H_3_ receptor (H_3_R) is a class A G protein‐coupled receptor (GPCR) that regulates neurotransmitter release in the central nervous system. Although structures of H_3_R in both inactive and active states have been elucidated, the functional roles of specific residues remain unclear. We previously identified four point mutations—L73^2.43^M, F193^ECL2^S, S359^6.36^Y, and C415^7.56^R—that restore signaling in yeast, where wild‐type H_3_R is otherwise inactive. Here, we show that these mutations also enhance constitutive (ligand‐independent) activity. All six possible double mutants exhibited markedly increased basal activity, indicating that these mutations act cooperatively to shift the conformational equilibrium toward the active state. In mammalian cells, all single mutations also increased constitutive activity compared with the wild‐type receptor. Additional mutagenesis at and around the four sites revealed that activity restoration cannot be explained solely by steric clashes introduced by residue substitution. Radioligand binding assays showed minimal changes in histamine affinity, implying that the mutations affect receptor function through conformational modulation. FSEC and FSEC‐TS analyses further demonstrated that increased constitutive activity correlates with reduced structural stability. When the same mutations were introduced into the histamine H_1_ receptor (H_1_R), only the C471^7.56^R mutant enhanced signaling, suggesting that the functional consequences of these mutations are receptor dependent. Collectively, our findings reveal a close relationship between structural destabilization and constitutive activation in H_3_R, while also underscoring the complexity of GPCR activation.

## 1 INTRODUCTION

G protein‐coupled receptors (GPCRs) constitute the largest protein family in humans and represent the most prominent class of drug targets (Rask‐Andersen et al., 2011). Located on the cell membrane, GPCRs transmit extracellular signals into the cell by undergoing conformational changes upon ligand binding, which in turn activates intracellular heterotrimeric G proteins. Once activated, these G proteins dissociate into α and βγ subunits, each of which modulates distinct downstream effectors, thereby triggering a wide range of intracellular signaling pathways. Elucidating the function of these key membrane proteins, which act as central hubs for extracellular signaling, provides crucial insights into disease mechanisms and facilitates drug discovery efforts.

The histamine H_3_ receptor (H_3_R) is a Class A GPCR predominantly expressed in the frontal regions of the central nervous system (Nieto‐Alamilla et al., 2016). H_3_R functions as both an autoreceptor and a heteroreceptor that suppresses the release of various neurotransmitters (Arrang et al., 1983). It has been implicated in the regulation of appetite, attention‐deficit hyperactivity disorder (ADHD), schizophrenia, cerebral ischemia, Parkinson's disease, and Alzheimer's disease, making it a promising therapeutic target for these neurological disorders (Peng et al., 2023; Yan et al., 2014; Yoshikawa et al., 2021). Currently, pitolisant, approved for the treatment of narcolepsy, remains the only marketed drug that targets H_3_R (Yuan et al., 2020), highlighting the considerable potential for further drug development. In recent years, structural insights into H_3_R have been obtained through the crystal structure of the receptor in complex with the antagonist PF‐03654746 (Peng et al., 2022), as well as cryo‐electron microscopy (cryo‐EM) structures of the receptor bound to histamine or other agonists together with the heterotrimeric Gi protein (Shen et al., 2024; Zhang et al., 2024). However, even these structural data do not provide a complete understanding of the activation mechanism of H_3_R.

Constitutively active mutations (CAMs) in GPCRs are known to promote agonist‐independent receptor activation and are often associated with disease phenotypes or altered drug sensitivities (Bond & Ijzerman, 2006; Parma et al., 1993; Parnot et al., 2002; Rosenberg Jr. et al., 2019; Schipani et al., 1995). Nevertheless, the molecular mechanisms by which CAMs enhance constitutive activity remain incompletely understood. H_3_R is inherently characterized by a relatively high level of constitutive activity (Morisset et al., 2000). Previous studies have shown that the hH_3_R(365) variant, in which 80 residues of intracellular loop 3 (ICL3) are deleted, displays elevated basal activity (Bongers et al., 2007), and that substitution of A357^6.34^ with lysine also increases constitutive activation (Takahashi et al., 2003). Beyond these findings, the structural determinants governing H_3_R constitutive activity remain largely unknown.

Yeast has been utilized as a model system for studying human GPCRs for more than a quarter of a century (King et al., 1990). Owing to its rapid growth and ease of genetic manipulation, *Saccharomyces cerevisiae* is well suited for high‐throughput experiments and has served as a valuable platform for protein engineering aimed at GPCR structural analysis (Shiroishi et al., 2012). In contrast to mammalian cells, which simultaneously express numerous GPCRs and multiple isoforms of G proteins (e.g., 16 Gα, 4 Gβ, and 12 Gγ subunits), yeast offers a simplified and well‐defined signaling environment. This simplicity enables precise analysis of the interaction between a single human GPCR and a specific G protein. Accordingly, yeast strains engineered to express chimeric G proteins that can couple with human GPCRs have been widely used for functional studies of various receptors (Deichmann et al., 2024; Kapolka et al., 2024). Moreover, yeast has played a pivotal role in the development of engineered GPCRs for chemogenetic applications in neuroscience, including receptors activated solely by synthetic ligands (RASSLs) and designer receptors exclusively activated by designer drugs (DREADDs) (Armbruster et al., 2007; Conklin et al., 2008). Despite its utility, only a subset of human GPCRs has been functionally characterized in yeast. Many GPCRs either fail to express or lose functional activity in the yeast system, highlighting the need for further optimization and investigation.

**FIGURE 1:**
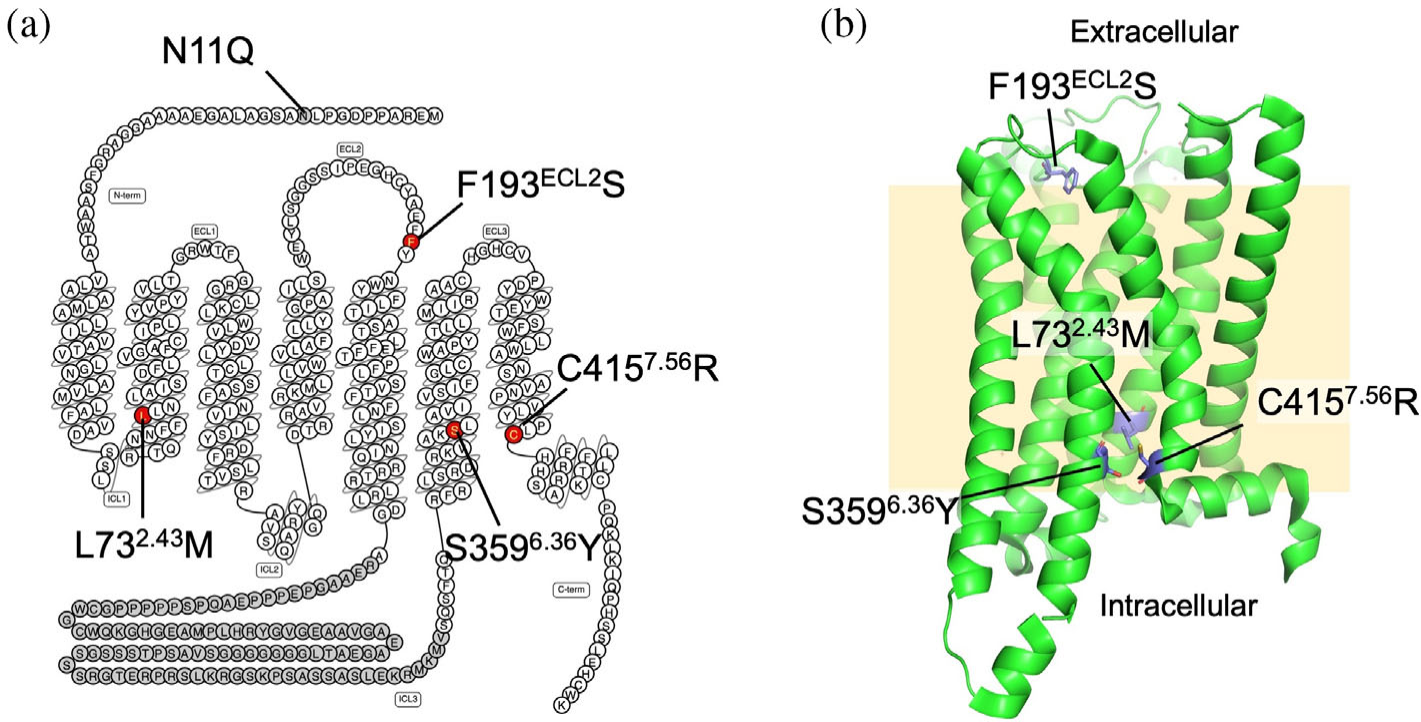
(a) Snake plot of H_3_R. The N11Q mutation, which prevents glycosylation, was introduced in all receptor constructs. The activity‐restoring mutations are shown in red. Residues shown in gray represent the third intracellular loop deleted in the H_3_R_i3d construct. (b) Backbone structure of the inactive‐state H_3_R (PDB ID: 7F61) shown as a green cartoon. Activity‐restoring mutations are indicated as purple sticks.

We previously identified four mutations—L73^2.43^M, F193^ECL2^S, S359^6.36^Y, and C415^7.56^R—that restore the signaling activity of H_3_R in *S. cerevisiae*, where the wild‐type receptor is otherwise nonfunctional (Watanabe et al., 2023). According to the classification proposed by Zhou et al., the transmembrane region of GPCRs can be divided into four distinct layers (Zhou et al., 2019). Among the four activity‐restoring mutations, three are located in the bottom‐most layer of the transmembrane domain, while one resides in the second extracellular loop (ECL2) (Figure 1a,b). Structural analysis of the H_3_R–Gi complex indicates that L73^2.43^, S359^6.36^, and C415^7.56^ are not directly involved in interactions with the heterotrimeric G protein. These mutations do not alter the G protein coupling specificity of H_3_R, and in the case of the C415^7.56^R mutant, ligand binding profiles—specifically for agonists and antagonists—remain largely unchanged (Watanabe et al., 2023). To date, there have been no detailed studies examining the functional roles of these four specific residues. In this study, we conducted a comprehensive functional and biochemical analysis of the four activity‐restoring mutations. We first generated double mutants and evaluated their signaling activity in *S. cerevisiae* using a yeast growth assay. We then assessed the binding affinity of each mutant for histamine using radiolabeled ligand binding assays. In addition, we evaluated the stability of the receptor variants using fluorescence‐detection size‐exclusion chromatography (FSEC) and thermal stability shift assays (FSEC‐TS). Finally, we introduced the corresponding mutations into the histamine H_1_ receptor (H_1_R), a related receptor subtype, to determine whether similar effects on signaling activity could be observed.

## 2 MATERIALS AND METHODS

### 2.1 The yeast expression vectors

We used the plasmid p416GPD_H_3_R_i3d‐GFP, previously described in our study (Watanabe et al., 2023), as the expression vector for H_3_R. In this construct, codon‐optimized *H*_*3*_ *R_i3d* is placed downstream of the constitutive GPD promoter and is followed by a TEV protease cleavage site, green fluorescent protein (GFP), and a C‐terminal histidine tag. The H_3_R_i3d variant lacks the third intracellular loop (ICL3; residues 237–340) and carries an N11Q mutation to prevent N‐linked glycosylation. We confirmed that this mutation does not affect receptor function, as the reverse mutation (Q11N), similar to H_3_R_i3d, failed to restore activity (Figure S1). A similar construct, p416GPD_H_1_R_i3d‐GFP, was generated for H_1_R based on the yeast expression vector for H_1_R_i3d used in our previous study (Shiroishi et al., 2012). In this variant, the N‐terminal 19 amino acids are deleted, and the ICL3 (residues 229–397) is removed.

### 2.2 Yeast strains and media

The yeast strains YB1, YB11, and YB14 were used for the growth assays (Dong et al., 2010). These strains express chimeric Gα proteins that couple to Gi, Gq, and Gs, respectively. The −Ura and −Ura−His dropout supplements were prepared according to a published formulation (Kaiser et al., 1994). YPAD medium (20 g/L peptone, 10 g/L yeast extract, 2% glucose, 20 mg/L adenine sulfate) was used for non‐selective cultivation. For selective conditions, synthetic complete (SC) medium lacking uracil (−Ura/SC; 1.7 g/L yeast nitrogen base without amino acids, 5 g/L ammonium sulfate, 1.92 g/L −Ura dropout supplement, and 2% glucose) or lacking both uracil and histidine (−Ura−His/SC; 1.7 g/L yeast nitrogen base without amino acids, 5 g/L ammonium sulfate, 1.92 g/L −Ura−His dropout supplement, and 2% glucose) was used. The *Escherichia coli* strain DH5α was cultivated in LB medium supplemented with 100 μg/mL ampicillin for plasmid preparation.

### 2.3 Site‐directed mutagenesis

Mutagenesis was performed using inverse PCR based on the protocol provided with the KOD‐Plus‐Neo Mutagenesis Kit (TOYOBO, Japan). Briefly, Dpn I was added to the PCR reaction mixture and incubated at 37°C for 1 h. The Dpn I‐treated PCR products were then ligated using T4 Polynucleotide Kinase (TOYOBO, Japan) and Ligation‐High (TOYOBO, Japan) at 16°C for 1 h. The ligation mixture was transformed into *E. coli* DH5α, plated onto LB agar containing ampicillin, and incubated at 37°C for 16 h. Plasmids were extracted from individual colonies and the presence of the intended mutations was confirmed by DNA sequencing.

### 2.4 Ligand concentration‐dependent yeast growth assay

Yeast colonies were cultured in 5 mL of −Ura/SC medium in test tubes and incubated at 30°C with shaking at 160 rpm for 20–22 h. Cells were harvested by centrifugation at 8000×*g* for 3 min, and the supernatant was discarded. The cell pellet was washed once with sterile water, centrifuged again at 8000×*g* for 3 min, and the supernatant was removed. The cells were then resuspended in −Ura−His/SC medium to an OD_600_ of 1.0. The suspension was further diluted to an OD_600_ of 0.02 in 500 μL of −Ura−His/SC medium containing 10–40 mM 3‐amino‐1,2,4‐triazole (3‐AT) and a fivefold serial dilution of ligands in a 96‐well deep‐well plate. The cultures were incubated at 25°C for 70 h at 1450 rpm using an M/BR‐022UP shaker (TAITEC, Japan). After incubation, 100 μL of each culture was transferred to a 96‐well microplate, and the OD_595_ was measured using an iMark microplate reader (Bio‐Rad, USA). Dose–response curve fitting was performed using GraphPad Prism (Dotmatics, USA).

### 2.5 Yeast membrane preparation

Yeast colonies were inoculated into 10 mL of –Ura/SC medium in test tubes and cultured at 30°C for 20–22 h with shaking at 160 rpm. The culture was then diluted to an OD_600_ of 0.12 in 16 wells of a 96‐deep‐well plate, each containing 1 mL of fresh –Ura/SC medium, and grown at 30°C for 20 h at 1450 rpm. Cells were harvested by centrifugation at 6000×*g* for 10 min at 4°C and resuspended in 700 μL of resuspension buffer (50 mM Tris–HCl pH 7.5, 5 mM EDTA, 10% glycerol, 0.12 M sorbitol) containing a 1× concentration of EDTA‐free complete protease inhibitor cocktail (Merck). For cell disruption, 500 μL of 0.5 mm glass beads were added to the cell suspension, and the mixture was subjected to vortexing at 4 °C for 10 min. Undisrupted cells and debris were removed by centrifugation at 3000×*g* for 30 min at 4°C. The resulting supernatant, containing the membrane fraction, was collected into microcentrifuge tubes and centrifuged at 100,000×*g* for 30 min at 4°C. The membrane pellet was resuspended in membrane buffer (50 mM Tris–HCl pH 7.5, 120 mM NaCl, 20% glycerol) supplemented with protease inhibitors as described above. Protein concentration was determined using the BCA Protein Assay Kit (Takara Bio, Japan).

### 2.6 In‐gel fluorescence

**FIGURE 2:**
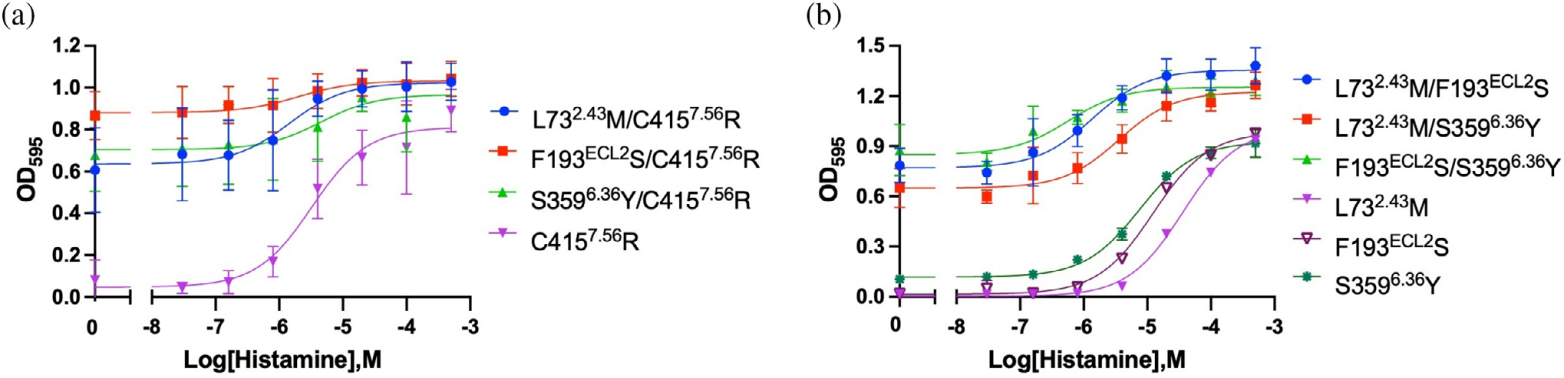
Histamine dose–response growth curves of H_3_R double mutants. (a) Growth curves for the C415^7.56^R single mutant and double mutants combining C415^7.56^R with each of the other three activity‐restoring mutations. (b) Growth curves for the L73^2.43^M, F193^ECL2^S, and S359^6.36^Y single mutants and their corresponding double mutants. Experiments were conducted using the YB1 yeast strain. Each data point represents the mean ± SD from three independent experiments, each performed in triplicate. Growth assay data were fitted using a nonlinear regression model (log[agonist] vs. response). Mean EC_50_ values are provided in Table 1.

**FIGURE 3:**
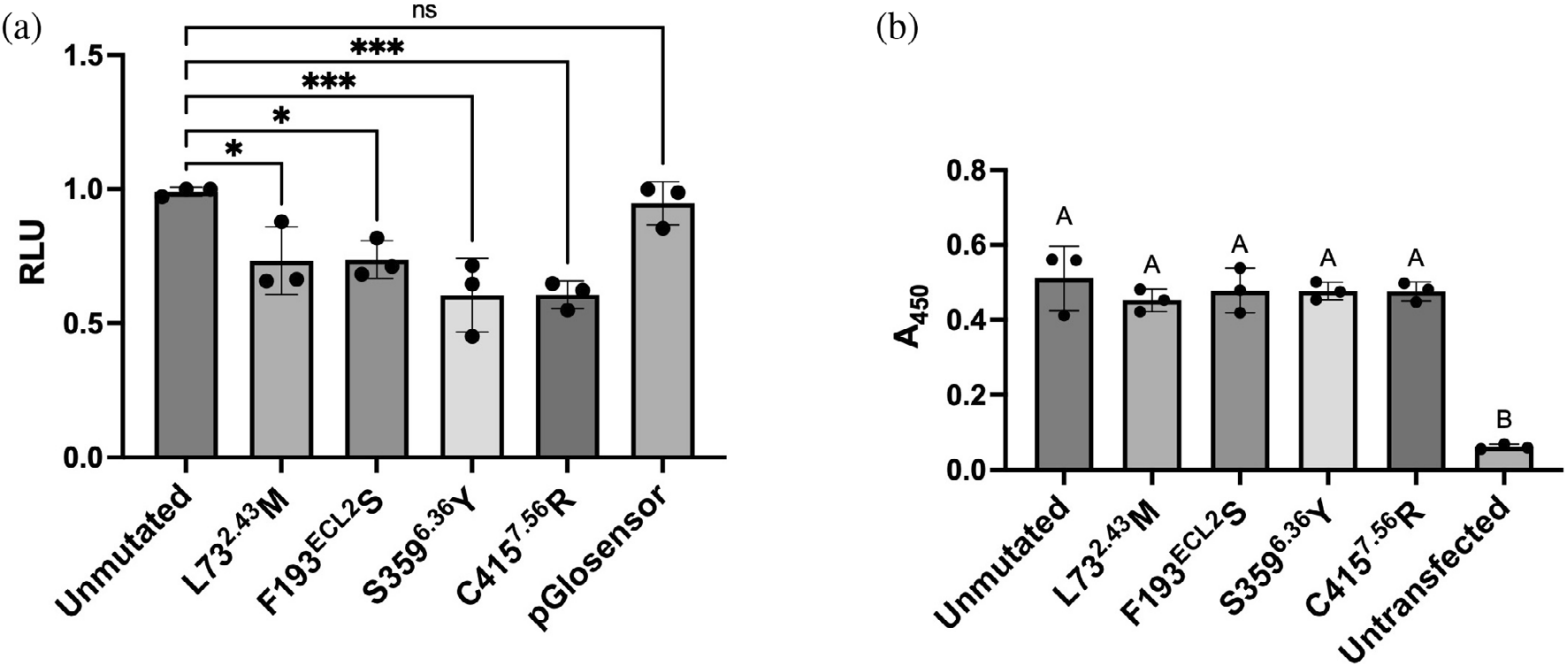
Constitutive activity of H_3_R mutants measured in mammalian cells. (a) Luminescence signals obtained using the pGloSensor™ system in the presence of D‐luciferin, showing inhibition of forskolin‐induced cAMP accumulation by unmutated H_3_R or its mutants in the absence of agonist. Statistical analysis was performed by one‐way ANOVA comparing unmutated H_3_R with each mutant. Asterisks indicate statistical significance (*p < 0.05, ***p < 0.001), and ns denotes not significant. (b) Cell‐surface expression of H_3_R determined by ELISA using an N‐terminal FLAG‐tag. Data are shown as the mean ± SD from three independent experiments. Different letters indicate statistically significant differences (one‐way ANOVA, *p* < 0.0001). All sample groups are labeled “A”, whereas the control group (untransfected cells) is labeled “B”, indicating that unmutated receptor and all mutants differ significantly from the control but not from each other.

Yeast membrane suspensions from the wild‐type and mutant receptors were adjusted to a protein concentration of 5 mg/mL. Fifteen microliters of each membrane suspension (5 mg/mL) were mixed with an equal volume of 2× sample buffer (50 mM Tris–HCl pH 7.5, 5 mM EDTA, 5% β‐mercaptoethanol, 5% glycerol, 4% SDS, 0.02% bromophenol blue, and a 2× concentration of complete EDTA‐free protease inhibitor cocktail). A total of 20 μL of the resulting mixture was loaded onto an SDS‐PAGE gel using the Tris–glycine buffer system, without boiling the samples. One microliter of Benchmark Fluorescent Protein Standard (Thermo Fisher Scientific, USA) was loaded as a molecular weight marker. Electrophoresis was carried out at 100 V and 4°C. Fluorescence images were acquired using a Typhoon FLA 7000 scanner (GE Healthcare, USA). In‐gel fluorescence images of the yeast expression of the mutants used in this study are shown in Figures 2 and 3.

### 2.7 Expression of H_3_R mutants in insect cells

A recombinant plasmid was constructed using the pFastBac1 vector, designed to encode a hemagglutinin (HA) signal peptide at the N‐terminus, and flanking the multiple cloning site (MCS), at the C‐terminus, a TEV protease recognition site, green fluorescent protein (GFP), and an octahistidine (8×His) tag. The H_3_R_i3d variant 2 (H_3_R_i3d(2))—featuring an N‐terminal deletion of 12 residues, deletion of the third intracellular loop (ICL3; residues 242–340), a C428S point mutation, and a C‐terminal deletion of 12 residues—was cloned into the vector. Site‐directed mutagenesis by inverse PCR was used to generate four H_3_R_i3d(2) mutants: L73^2.43^M, F193^ECL2^S, S359^6.36^Y, and C415^7.56^R. Recombinant bacmid DNA was generated using DH10Bac *E. coli* cells (Thermo Fisher Scientific, USA), following the manufacturer's instructions. Sf9 insect cells (Thermo Fisher Scientific, USA) were transfected with the bacmid DNA at a density of 3.3 × 10^5^ cells/mL in a 24‐well plate using ExpiFectamine reagent (Thermo Fisher Scientific, USA). The P0 viral stock was harvested 7 days post‐transfection. For virus amplification, 200 μL of P0 virus was added to 2 mL of Sf9 cells seeded at a density of 6.6 × 10^5^ cells/well in a 6‐well plate. The culture was incubated at 27°C for 6 days, and the resulting P1 viral stock was collected and stored at 4°C. For protein expression, 50 μL of P1 virus was added to 10 mL of Sf9 cells at a density of 3.3 × 10^6^ cells/mL in a 25 mL Erlenmeyer flask. The culture was incubated at 27°C with shaking at 125 rpm. Cells were harvested at peak GFP fluorescence (approximately 72 h post‐infection), collected by centrifugation, flash‐frozen in liquid nitrogen, and stored at −80°C until use.

### 2.8 Membrane preparation from insect cells

Insect cells were lysed and resuspended in 3 mL of 1× phosphate‐buffered saline (PBS) supplemented with a 1× concentration of Complete Protease Inhibitor EDTA‐free Cocktail (Roche, Switzerland) (1× PI). Cell disruption was performed carefully by sonication, and the extent of lysis was monitored microscopically. After lysis, the suspension was subjected to ultracentrifugation at 40,000 rpm for 30 min at 4°C to isolate the membrane fraction. The resulting membrane pellet was resuspended in 2 mL of membrane buffer (50 mM Tris–HCl pH 7.5, 120 mM NaCl, 20% glycerol) containing 1× PI, aliquoted, and stored at −80°C until use.

### 2.9 Radioisotope‐labeled ligand binding assay

The membrane suspension, [^3^H]‐histamine (PerkinElmer, USA), and unlabeled histamine were diluted in assay buffer (20 mM HEPES, pH 7.5, 150 mM NaCl). Briefly, approximately 300 μg of membrane protein was included in each 100 μL reaction mixture, which was dispensed in triplicate into a 96‐well microplate. The final concentrations of [^3^H]‐histamine were 200, 100, 50, 25, 12.5, 6.25, 3.13, and 1.56 nM. To assess nonspecific binding, parallel reactions were prepared with unlabeled histamine at a 1000‐fold excess relative to the radiolabeled ligand. The reaction mixtures were incubated at room temperature (22–24°C) for 1 h. Membranes were collected on Filtermat B glass fiber filters (PerkinElmer, USA) pre‐soaked in 0.3% polyethyleneimine, using a FilterMate cell harvester (PerkinElmer, USA). The filters were washed with distilled water, dried, and overlaid with MeltiLex B/HS solid scintillator (PerkinElmer, USA), which was melted onto the filter. Radioactivity was measured using a MicroBeta2 microplate scintillation counter (PerkinElmer, USA). Nonlinear curve fitting (One site‐specific binding) was performed using GraphPad Prism to calculate the dissociation constant (*K*_d_). *K*_d_s from three to four independent experiments were compared by statistical analysis. Log‐transformed *K*_d_ values were analyzed using one‐way ANOVA, and no significant differences were observed among unmutated and mutant receptors.

### 2.10 Fluorescence‐detection size‐exclusion chromatography

For solubilization, 50 μL of membrane suspension was mixed with 50 μL of resuspension buffer (50 mM Tris–HCl, pH 7.5, 5 mM EDTA, 10% glycerol, 0.12 M sorbitol) containing 1% n‐dodecyl‐β‐D‐maltoside (DDM) and 0.2% cholesterol hemisuccinate (CHS). The mixture was incubated on ice for 1 hour. Following solubilization, the sample was centrifuged at 50,000 rpm for 30 min at 4°C to remove insoluble material. The resulting supernatant was subjected to FSEC. FSEC was performed using an HPLC system (Shimadzu, Japan) equipped with a Sepax SRT SEC‐300 column 4.6 × 150 mm (Sepax Technologies, USA). The flow rate was set to 0.35 mL/min. Fluorescence detection was performed with an excitation wavelength of 490 nm and an emission wavelength of 525 nm. The mobile phase consisted of GF buffer (20 mM Tris–HCl, pH 7.5, 150 mM NaCl, 0.03% DDM, 0.006% CHS). A gel filtration standard (Bio‐Rad, USA) was used as a molecular weight marker.

For thermal stability analysis, membrane proteins were solubilized and ultracentrifuged as described above. The resulting supernatant was aliquoted into 100 μL portions and incubated at temperatures ranging from 20 to 80°C, in 10°C increments, for 10 min. After heat treatment, samples were subjected to FSEC analysis as described in the previous section. Fluorescence intensity at the monomer elution time was quantified for each temperature condition and plotted against temperature. Nonlinear curve fitting (Boltzmann sigmoidal) was performed using GraphPad Prism to estimate thermal stability.

### 2.11 GloSensor cAMP assay

The constitutive activity of H_3_R was assessed by measuring the inhibition of forskolin‐induced cAMP production using the pGloSensor™‐22F cAMP Assay (Promega, USA). An H_3_R variant generated from H_3_R_i3d(2) by reverting Gln11 to the native Asn was expressed in Expi293F cells by transient transfection using the pcDNA3.4 vector. Cells were co‐transfected with the pGloSensor™‐22F plasmid and the respective H_3_R construct at a 1:1 weight ratio using PEI MAX (Polysciences, Inc., USA). One day post‐transfection, cells were harvested by centrifugation at 300×*g* for 5 min and washed with HE200 medium. The cell pellets were resuspended in HE200 medium containing D‐luciferin potassium salt solution (final 2 mM). The cell suspension was seeded into a 96‐well white plate at 45 μL per well and incubated for 2 h at room temperature to facilitate substrate loading. Following this incubation, forskolin was added to a final concentration of 20 μM. After a further 30‐min incubation at room temperature, luminescence was measured using a 1420 ARVO Light luminescence counter (PerkinElmer, USA).

Relative cell surface expression of the N‐terminally FLAG‐tagged H_3_R constructs was quantified by a whole‐cell ELISA performed in 1.5 mL microfuge tubes. Transfected cells were washed with cold PBS by centrifugation (300×*g*, 5 min, 4°C). The cell pellet was resuspended in 100 μL of HRP‐conjugated anti‐FLAG antibody (ab49763, Abcam, UK), diluted 1:2500 in 1% BSA in PBS, and incubated at 4°C for 1 h. After incubation, the cells were washed three times with 0.2 mL of cold PBS via centrifugation. The final cell pellet was resuspended in 100 μL of TMB (3,3′,5,5′‐tetramethylbenzidine) substrate working solution, prepared immediately before use. The reaction was allowed to proceed for 20 min at room temperature, protected from light, and then stopped by the addition of 100 μL of 1 M HCl. After pelleting the cells by centrifugation, 200 μL of the colored supernatant was transferred to a clear, flat‐bottom 96‐well plate. The absorbance was measured at 450 nm using an iMark microplate reader (Bio‐Rad, USA).

## 3 RESULTS AND DISCUSSION

### 3.1 Enhanced constitutive signaling activity by double combinations of activity‐restoring mutations

Although wild‐type H_3_R does not exhibit signaling activity in yeast, we previously identified four mutations—L73^2.43^M, F193^ECL2^S, S359^6.36^Y, and C415^7.56^R—through random mutagenesis and screening, each of which restored ligand‐dependent activity when introduced individually into H_3_R (Watanabe et al., 2023). We hypothesize that H_3_R adopts an inactive conformation in yeast, and that these mutations promote a shift toward the active state. However, the interplay between these four activity‐restoring mutations remains unclear. To investigate potential synergistic effects, we generated six double mutants by combining all possible pairs of the four mutations, and evaluated their signaling activity using ligand (histamine)‐dependent yeast growth assays. All six double mutants exhibited markedly increased constitutive activity compared to the single mutants, and in several of these double mutants a decrease in EC_50_ values was also observed (Figure 2a,b and Table 1). These results indicate that the activity‐restoring mutations act cooperatively to increase the propensity of H_3_R to adopt an active conformation.

**TABLE 1:** EC_50_ values of H_3_R double mutants determined by yeast‐based growth assay.

Mutant	EC_50_ [μM]
L73^2.43^M/C415^7.56^R	1.4 ± 0.5
F193^ECL2^S/C415^7.56^R	0.23 ± 0.05
S359^6.36^Y/C415^7.56^R	8.5 ± 6.4
C415^7.56^R	3.5 ± 1.5
L73^2.43^M/F193^ECL2^S	1.9 ± 0.5
L73^2.43^M/S359^6.36^Y	6.7 ± 2.1
F193^ECL2^S/S359^6.36^Y	1.0 ± 0.4
L73^2.43^M	36.3
F193^ECL2^S	12.4
S359^6.36^Y	7.5

*Note*: Values represent the mean ± SD from three independent experiments performed in triplicate, except for the L73^2.43^M, F193^ECL2^S, and S359^6.36^Y mutants, which were assessed in a single experiment.

Next, we examined how these four mutations influence H_3_R function in mammalian cells. Compared with the unmutated receptor, all four mutants exhibited higher levels of Gi activity in the absence of agonist, as evidenced by a greater reduction in luminescence in the pGloSensor assay (Figure 3a). Notably, cell‐surface expression levels did not differ significantly among the mutants and the unmutated receptor (Figure 3b). These results demonstrate that, in mammalian cells, these activity‐restoring mutations enhance the constitutive activity of H_3_R.

### 3.2 Effect of alternative amino acid substitutions on activity restoration

**FIGURE 4:**
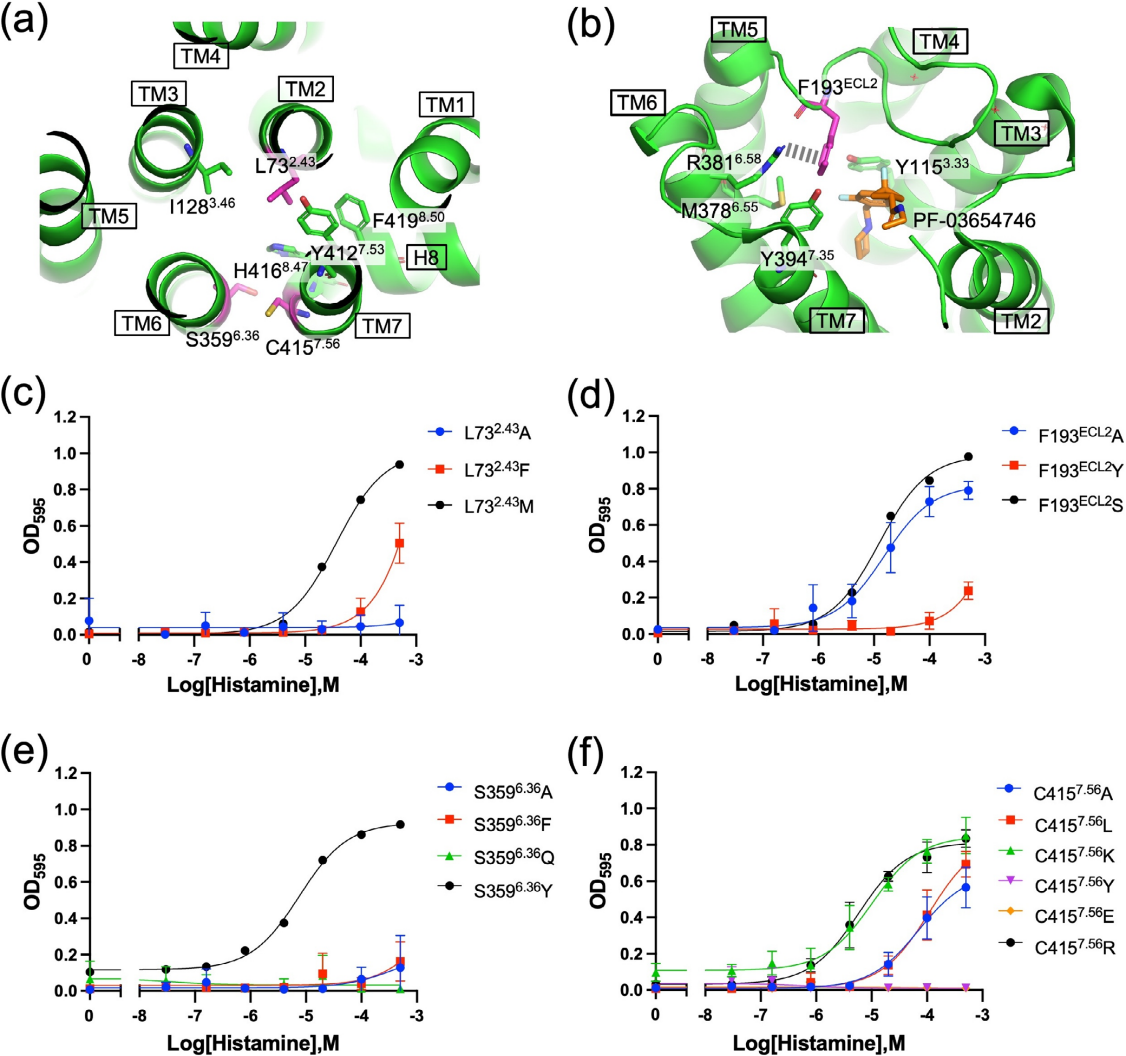
Structural context and histamine dose–response growth curves for H_3_R mutants. (a,b) Close‐up views of the wild‐type H_3_R structure (PDB ID: 7F61) highlighting the local environments of the indicated residues. In (a), L73^2.43^, S359^6.36^, and C415^7.56^ are shown in magenta; in (b), F193^ECL2^ is shown in magenta. The ligand PF‐03654746 is shown in orange. Putative cation–π interactions are indicated by thick gray dashed lines. (c–f) Histamine dose–response growth curves of H_3_R mutants at positions L73^2.43^ (c), F193^ECL2^ (d), S359^6.36^ (e), and C415^7.56^ (f). Experiments were conducted using the YB1 yeast strain. Each data point represents the mean ± SD from three independent experiments, each performed in triplicate, except for the L73M, F193S, and S359Y mutants, for which only a single experiment was conducted. Growth assay data were fitted using a nonlinear regression model (log[agonist] vs. response). Mean EC_50_ values are provided in Table 2.

According to the inactive‐state crystal structure of H_3_R (PDB ID: 7F61), three of the four activity‐restoring mutations—L73^2.43^M, S359^6.36^Y, and C415^7.56^R—are located in the bottom layer of the transmembrane region (Figure 1b). S359^6.36^ and C415^7.56^ interact with each other, while L73^2.43^ does not interact with these residues but with residues in TM3 and TM7 (Figure 4a and Table S1). All three involve substitutions with bulkier side chains. When the residue substitutions were modeled in PyMOL, steric clashes with surrounding residues were observed in 12 out of 13 possible sidechain rotamers for L73^2.43^M. In addition, both S359^6.36^Y and C415^7.56^R exhibited clashes with surrounding residues in all rotamers. Therefore, these mutations may induce steric clashes within the receptor core directly or may induce local strain, particularly when combined in double mutants, thereby favoring a shift toward the active conformation and enhancing constitutive activity. F193^ECL2^ is located in the second extracellular loop (ECL2) and interacts with Y115^3.33^, M378^6.55^, R381^6.58^, Y394^7.35^ and the ligand (Figure 4b). The contact with Y115^3.33^ involves only the hydroxyl group of the tyrosine side chain and does not appear to be critical. In contrast, F193^ECL2^ forms a cation–π interaction with R381^6.58^, forms edge‐to‐face π–π stacking with Y394^7.35^ and makes hydrophobic contacts with M378^6.55^ (Table S1). In contrast to three other activity‐restoring mutants, F193^ECL2^S results in a reduction in side chain size. The replacement of phenylalanine with serine likely disrupts aromatic interactions with nearby residues, which may destabilize the inactive conformation and facilitate activation. These observations suggest that interactions between F193^ECL2^ and its neighboring residues play a role in maintaining the inactive state of H_3_R.

To further investigate how side chain size influences signaling activity, we introduced additional substitutions at each of the four positions using amino acids with varying side chain volumes, and examined their activity using ligand‐dependent yeast growth assays (Figure 4c–f and Table 2). At position L73^2.43^, substitution with phenylalanine (larger than leucine or methionine) resulted in modest activity, whereas alanine substitution failed to restore signaling activity. At position F193^ECL2^, alanine substitution (F193^ECL2^A) exhibited activity comparable to that of serine, while tyrosine substitution resulted in minimal activity. These results support our hypothesis that smaller side chains at this position reduce interactions with surrounding residues and promote receptor activation. At position S359^6.36^, substitutions with alanine, phenylalanine, or glutamine did not restore activity, suggesting that not only side chain volume but also polarity may be important at this site. At position C415^7.56^, substitution with lysine (C415^7.56^K) produced a level of activity comparable to that of the arginine mutant. Substitutions with alanine or leucine led to modest activity restoration, while substitutions with tyrosine or glutamic acid were inactive. These findings suggest that a positively charged residue at position 7.56 may be critical for activity restoration.

**TABLE 2:** EC_50_ values of H_3_R single‐point mutants at positions L73^2.43^, F193^ECL2^, S359^6.36^, and C415^7.56^ determined by yeast‐based growth assay.

Mutant	EC_50_ [μM]	Mutant	EC_50_ [μM]
L73^2.43^M	36.3	C415^7.56^R	4.0 ± 1.6
L73^2.43^A	ND	C415^7.56^A	49 ± 10
L73^2.43^F	ND	C415^7.56^K	10.8 ± 5.0
F193^ECL2^S	12.4	C415^7.56^E	ND
F193^ECL2^A	17.8 ± 6.4	C415^7.56^L	148 ± 65
F193^ECL2^Y	ND	C415^7.56^Y	ND
S359^6.36^Y	7.5		
S359^6.36^A	ND		
S359^6.36^F	ND		
S359^6.36^Q	ND		

*Note*: Values represent the mean ± SD of three independent experiments, except for the L73^2.43^M, F193^ECL2^S, and S359^6.36^Y mutants, which were the same data as Table 1. ND, not determined.

Substitution of L73^2.43^ with bulkier residues was expected to create steric clashes with surrounding residues, thereby facilitating activation, whereas substitution of F193^ECL2^ with smaller residues was thought to reduce stabilizing interactions, which might also promote activation. To test these possibilities, we introduced mutations into neighboring residues that interact with L73^2.43^ and F193^ECL2^. For I128^3.46^ and Y412^7.53^, which interact with L73^2.43^ (Figure 4a), we generated bulkier variants (I128^3.46^M and Y412^7.53^W) to induce steric clashes with L73^2.43^. These were combined with the distant F193^ECL2^S mutation to examine whether they further enhanced activity. Similarly, for residues surrounding F193^ECL2^, R381^6.58^, and Y394^7.35^, which are thought to form strong interactions with F193^ECL2^ (Figure 4b), were replaced with smaller residues (R381^6.58^A and Y394^7.35^A) and combined with the distant C415^7.56^R mutation. However, none of these combinations resulted in further enhancement of activity (Figure S4a–c).

These findings suggest that the effects of mutations at L73^2.43^ and F193^ECL2^ cannot be explained simply by steric clashes or loss of interactions with their immediate neighbors. Instead, they may perturb the cooperative rearrangements of transmembrane helices and the long‐range allosteric coupling that underlie GPCR activation. Similarly, the functional consequences of S359^6.36^Y and C415^7.56^R are unlikely to be attributable solely to substitutions with bulkier residues. Taken together, these results highlight the multifaceted nature of GPCR activation, where local steric effects are integrated with broader conformational dynamics.

### 3.3 Activity‐restoring mutations do not affect histamine binding affinity of H_3_R

**FIGURE 5:**
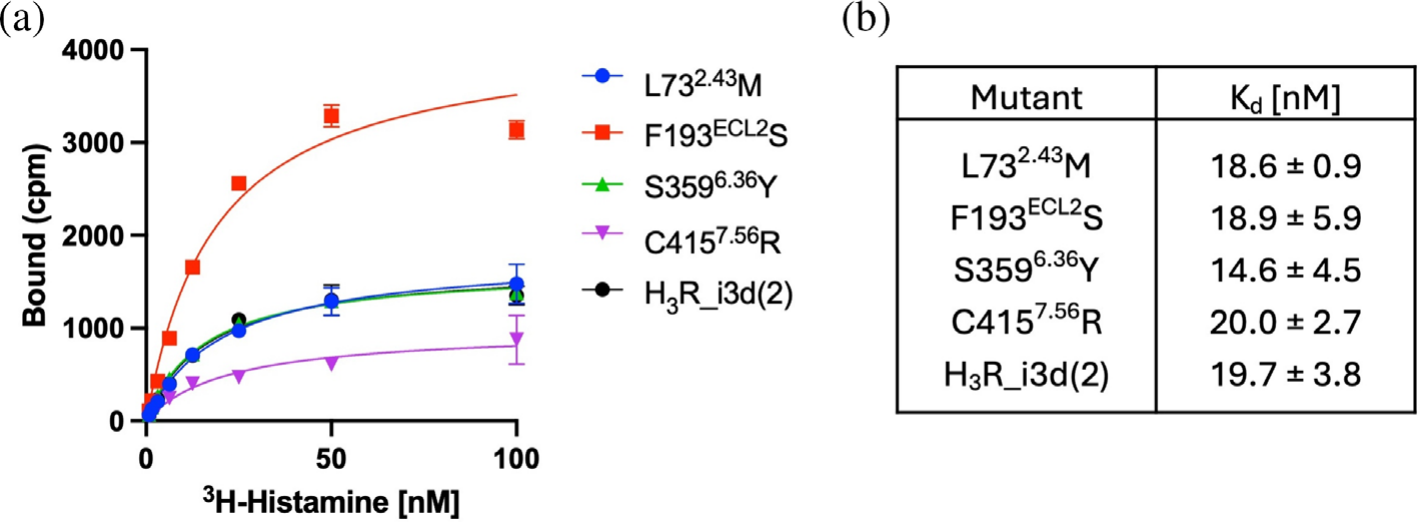
Saturation binding of [^3^H]‐histamine to unmutated H_3_R_i3d(2) and its mutants expressed in insect cells. (a) A representative curve from one of three independent experiments is shown. Data were fitted using a nonlinear regression model (one‐site specific binding). (b) Dissociation constants (*K*_d_) of [^3^H]‐histamine binding to unmutated and mutant H_3_R. Values represent the mean ± SD from three to five independent experiments. No significant differences were observed among groups.

In our previous study, we performed a [^3^H]‐histamine binding assay using membrane fractions prepared from *S. cerevisiae* expressing the H_3_R C415^7.56^R mutant (Watanabe et al., 2023). The results suggested that the mutant retained similar histamine binding to that of the wild‐type receptor. However, due to the low expression level of H_3_R in yeast, we were unable to determine the dissociation constant (*K*_d_). To overcome this limitation, we employed a baculovirus–insect cell expression system to express H_3_R. Each mutation was introduced into an engineered H_3_R variant, termed H_3_R_i3d(2), which differs from H_3_R_i3d in that five residues at the intracellular end of TM5—originally present in the native H_3_R sequence—were restored. In addition, H_3_R_i3d(2) includes an N‐terminal deletion of 12 residues, a C‐terminal deletion of 12 residues, and a C428S point mutation. Despite this structural difference, H_3_R_i3d(2) exhibited signaling activity in yeast nearly identical to that of H_3_R_i3d. For the four activity‐restoring mutants (L73^2.43^M, F193^ECL2^S, S359^6.36^Y, and C415^7.56^R), we carried out a saturation binding assay using [^3^H]‐histamine (Figure 5a,b). The dissociation constant of the H_3_R_i3d(2) (unmutated) for [^3^H]‐histamine was determined to be 19.7 ± 3.8 nM, which is comparable to values reported previously for H_3_R (Nguyen et al., 2001). Importantly, all four mutants exhibited *K*_d_ values similar to that of unmutated H_3_R_i3d(2), with no substantial changes observed, and statistical analysis confirmed that these differences were not significant. These results indicate that the activity‐restoring mutations do not significantly alter the affinity of H_3_R for histamine.

### 3.4 Activity‐restoring mutations destabilize the receptor

**FIGURE 6:**
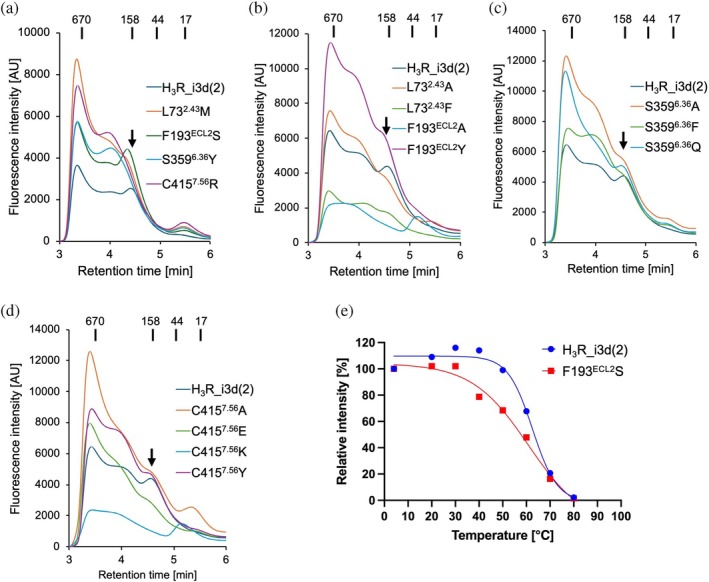
Stability assessment of H_3_R mutants. (a–d) FSEC chromatograms of detergent‐solubilized unmutated H_3_R_i3d(2) and mutants analyzed in this study in 1% DDM/0.2% CHS. The elution positions of molecular size markers and their corresponding molecular weights (×10^3^) are indicated at the top of each chromatogram. Arrows indicate the monomeric peak position of unmutated H_3_R_i3d(2). (e) FSEC‐TS results for unmutated H_3_R_i3d(2) and the F193^ECL2^S mutant. Fluorescence intensities at the monomer elution time were plotted for each temperature. The *T*_m_ values were 62.6°C for unmutated H_3_R_i3d(2) and 60.8°C for F193^ECL2^S.

The activity‐restoring mutations enhance the constitutive activity of H_3_R. Previous studies on the cannabinoid CB2 receptor and the adenosine A_2A_ receptor (A_2A_R) have demonstrated an inverse relationship between constitutive activity and receptor stability. In the case of CB2, this relationship was revealed through mutational analyses in transmembrane helices 2 and 3, where mutations that increased constitutive activity led to reduced thermal stability, while those that decreased constitutive activity enhanced stability (Ahn et al., 2013). Similarly, for A_2A_R, receptor variants stabilized in the active conformation exhibited greater thermal stability than the wild type, while displaying reduced constitutive activity (Bertheleme et al., 2013). To investigate whether the activity‐restoring mutations affect receptor stability, we introduced each mutation into an H_3_R_i3d(2) and analyzed it. We expressed unmutated H_3_R_i3d(2) and four mutant variants in insect cells, solubilized the membrane fractions using DDM/CHS, and analyzed them by fluorescence‐detection size‐exclusion chromatography (FSEC) with C‐terminal GFP used as a reporter. As shown in Figure 6a, clear peaks corresponding to the monomeric form of H_3_R (near the 158 kDa molecular weight marker) were observed for unmutated H_3_R_i3d(2) and the F193^ECL2^S mutant, although the peak for the F193^ECL2^S appeared slightly shifted toward the higher‐molecular‐weight side. In contrast, the other three mutants—L73^2.43^M, S359^6.36^Y, and C415^7.56^R—exhibited little to no monomeric peak, with a clear shift toward larger, higher molecular weight species indicative of oligomerization. In SEC analyses of membrane proteins, such shifts typically indicate destabilization of the receptor in detergent micelles, leading to aggregation or oligomer formation (Kaneko et al., 2024; Kean et al., 2015; Rejnowicz et al., 2023). These results suggest that, while monomeric forms of unmutated H3R_i3d(2) and the F193^ECL2^S mutant are maintained upon solubilization, the other three activity‐restoring mutants destabilize the receptor and promote oligomerization under these conditions. The mutants discussed in Figure 4c–f were also analyzed by FSEC. As a result, the mutants that showed activity restoration (F193^ECL2^A and C415^7.56^K) also exhibited loss of the monomeric peak (Figure 6b–d).

To further evaluate the thermal stability of unmutated H_3_R and the F193^ECL2^S mutant, we performed FSEC‐based thermal stability assays (FSEC‐TS). Solubilized receptors prepared at 4°C were incubated at temperatures ranging from 20 to 80°C for 10 min each, followed by SEC analysis (Figures 6e and S5). For the F193^ECL2^S mutant, a reduction in the monomer peak was observed at lower temperatures compared to the unmutated receptor, indicating that the mutation decreases the thermal stability of the receptor and promotes oligomerization at milder heat conditions. The F193^ECL2^S mutation abolishes multiple interactions formed by the phenylalanine residue, leading to destabilization of the receptor. Although the underlying mechanism is not fully understood, this mutation appears to increase the propensity of H_3_R to adopt an active state. F193^ECL2^, together with the adjacent F192^ECL2^, constitutes an FF motif that is conserved in the histamine H_4_ receptor (H_4_R). In H_4_R, substitution of these residues with valine or alanine greatly reduces constitutive activity, and the FF motif has been identified as a key determinant of the receptor's high basal activity (Wifling et al., 2015). Interestingly, this observation contrasts with our findings in H_3_R, where the F193^ECL2^S mutation enhanced constitutive activity. In a homology model of the inactive state of H_4_R obtained from the GPCRdb (version 2024‐05‐15) (Pandy‐Szekeres et al., 2023), F169^ECL2^ (corresponding to F193^ECL2^ in H_3_R) does not appear to form a meaningful interaction with TM6. This structural difference may underlie the distinct mechanisms by which constitutive activity is regulated in H_3_R and H_4_R.

### 3.5 Introduction of activity‐restoring mutations into H_1_R and their effects on signaling activity

**FIGURE 7:**
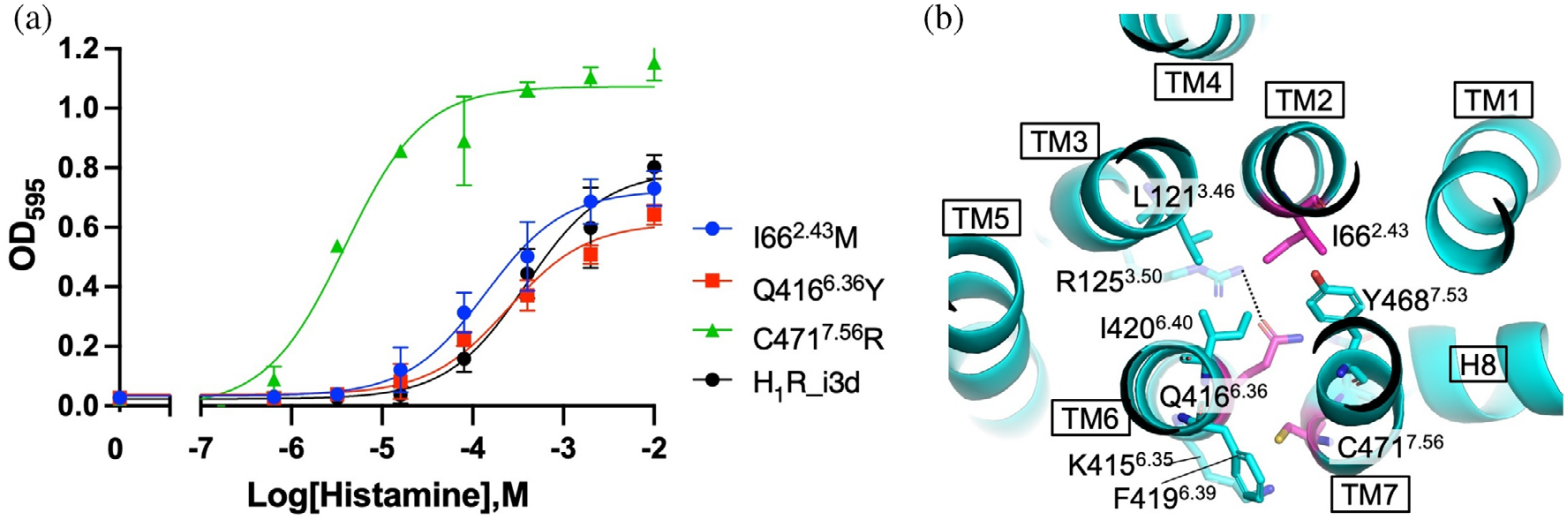
Effect of H_3_R‐derived mutations on H_1_R signaling. (a) Agonist (histamine) concentration‐dependent growth curves of unmutated H_1_R_i3d and its mutants. Experiments were conducted using the YB11 yeast strain. Each data point represents the mean ± SD from three independent experiments, each performed in triplicate. The growth assay data were fitted using a nonlinear regression model (log[agonist] vs. response). Mean EC_50_ values are provided in Table 3. (b) Close‐up views of the wild‐type H_1_R structure (PDB ID: 3RZE) highlighting the local environments of the indicated residues (L73^2.43^, S359^6.36^, and C415^7.56^), which are shown in magenta. Dashed line indicates a hydrogen bond.

While H_3_R is classified as a high‐affinity receptor for histamine, histamine H_1_ receptor (H_1_R) is considered a low‐affinity subtype. The amino acid sequence similarity between these receptors is relatively low (approximately 22%). Our previous study demonstrated that H_1_R displays signaling activity in yeast even in the absence of mutations (Watanabe et al., 2023). In this study, we investigated whether activity‐restoring mutations identified in H_3_R, particularly those located in the bottom layer (positions 2.43, 6.36, and 7.56), would have similar effects when introduced into another receptor subtype. H_1_R is known to preferentially couple to the Gq protein. We expressed a modified H_1_R variant lacking the third intracellular loop (H_1_R_i3d) in the YB11 yeast strain, which expresses a chimeric Gq, and performed ligand‐dependent growth assays (Figure 7a and Table 3). Among the three mutations tested, the C471^7.56^R mutant exhibited a marked enhancement in both potency (with an ~100‐fold decrease in EC_50_) and efficacy. In contrast, the I66^2.43^M and Q416^6.36^Y mutants showed no significant changes in activity compared to unmutated H_1_R_i3d. None of the H_1_R mutants exhibited ligand‐dependent growth when expressed in YB1 or YB14 yeast strains, which express chimeric Gi and Gs, respectively (Supplementary Figure 6), indicating that these mutations do not affect G protein selectivity in H_1_R.

**TABLE 3:** EC_50_ values of H_1_R mutants at positions L66^2.43^, Q416^6.36^, and C471^7.56^ determined by yeast‐based growth assay.

Mutant	EC_50_ [μM]	*n*
I66^2.43^M	142 ± 55	4
Q416^6.36^Y	250 ± 64	3
C471^7.56^R	5.0 ± 2.0	4
Unmutated	465 ± 164	4

*Note*: Values represent the mean ± SD from three or four independent experiments.

Position 2.43 is highly conserved among class A GPCRs and is typically occupied by leucine or isoleucine. While the L73^2.43^M mutation in H_3_R facilitated a shift toward the active state, the corresponding I66^2.43^M mutation in H_1_R had little effect. Comprehensive mutagenesis studies on the β_2_‐adrenergic receptor (β_2_AR) have identified the I72^2.43^ residue as a “connected driver,” important for receptor activation, particularly for potency (Heydenreich et al., 2023). Although H_1_R and H_3_R have similar residues at position 2.43 (Ile and Leu, respectively), their local environments differ (Figure 7b and Table S2), possibly explaining the distinct functional outcomes of the mutations. As for Q416^6.36^Y, the relatively minor change in activity may be due to the modest difference in side‐chain volume and the orientation of the Q416 side chain, which points inward and forms limited contacts with other residues (Figure 7b). In many aminergic receptors, position 6.36 is typically occupied by small side‐chain residues such as serine, threonine, or valine. In contrast, H_1_R has a larger polar residue, glutamine, at this position. In β_2_AR, position 6.36 has been classified as a “bystander” residue that is relatively insensitive to mutation (Heydenreich et al., 2023). Position 7.56 is typically cysteine in histamine receptors but is phenylalanine in many other aminergic GPCRs. Notably, there is no corresponding residue in β₂AR for position 7.56. C471^7.56^ interacts with residues in TM6 (Table S2). When a residue‐substitution model was generated based on the inactive‐state structure of H_1_R (PDB ID: 3RZE), steric clashes with surrounding residues were observed. This destabilization may facilitate activation of H_1_R.

## 4 CONCLUSION

In this study, we demonstrated that the four point mutations—L73^2.43^M, F193^ECL2S^, S359^6.36^Y, and C415^7.56^R—previously identified as restoring the function of the human histamine H_3_ receptor (H_3_R) in a yeast expression system, also significantly increase its constitutive activity. Each mutation independently restored activity; however, all possible combinations of double mutants showed a substantial increase in constitutive signaling, indicating a structural shift that favors the active conformation. Importantly, experiments in mammalian cells confirmed that these four mutants exhibit higher constitutive activity compared with the wild‐type receptor. Furthermore, experiments using FSEC and FSEC‐TS revealed that they significantly affected the structural stability of the receptor. In particular, three mutations located within the transmembrane domain (L73^2.43^M, S359^6.36^Y, and C415^7.56^R) reduced the stability of the monomeric receptor. The F193^ECL2^S mutation, situated in the FF motif of the second extracellular loop, lost aromatic interactions and thereby also decreased stability, facilitating the transition to the active state. This contrasts with the histamine H_4_ receptor (H_4_R), where the FF motif is essential for maintaining high constitutive activity, suggesting the existence of receptor‐specific mechanisms of activation. In addition to these four mutations, we identified two additional activity‐restoring mutations (F193^ECL2^A and C415^7.56^K). Like the original four, these mutants also showed disappearance of the monomeric peak in FSEC analysis. These findings support the conclusion that enhanced constitutive activity in H_3_R is closely linked to structural destabilization. Furthermore, these mutations promoted receptor activation without altering the binding affinity for histamine. Among the activity‐restoring mutations discovered in H_3_R, C415^7.56^R was particularly notable for its strong effect when introduced into H_1_R, where it significantly increased both potency and efficacy. This suggests that certain mutations can act in a conserved manner across GPCR subtypes, despite low overall sequence similarity. Conversely, the absence of effect at the same positions in some receptors highlights the importance of receptor‐specific structural contexts in determining mutational impact.

In the present study, we also found that, whereas single activity‐restoring mutations did not increase constitutive activity in the yeast expression system, they did enhance constitutive activity in the mammalian expression system. One limitation of the yeast expression system is the difference in membrane lipid composition compared to human cells. Notably, yeast membranes contain ergosterol instead of cholesterol. In both crystal and cryo‐EM structures of H_3_R, cholesterol binding is consistently observed between TM1 and TM7 (Peng et al., 2022; Shen et al., 2024; Zhang et al., 2024), suggesting that cholesterol may contribute to the functional regulation of H_3_R. If so, the lack of cholesterol in yeast membranes could partly explain the reduced receptor activity observed in this system. Additionally, several non–proton‐sensing GPCRs have been shown to be modulated by extracellular pH (Ghanouni et al., 2000; Kapolka et al., 2021; Meyer et al., 2019; Vogel et al., 2008). The medium used in this study had a slightly acidic pH (just below 6), raising the possibility that H_3_R activity may have been suppressed under these conditions. Further investigation will be needed to explore these hypotheses. Looking ahead, molecular dynamics simulations could be used to examine how these mutations affect the receptor's allosteric and water‐mediated networks. Moreover, the insights gained from H_3_R in this study may be extended to other GPCRs, potentially informing the design of artificial receptors and the development of next‐generation DREADDs. Finally, knowledge of mutations that modulate constitutive activity may aid in understanding pathogenic GPCR variants and contribute to the development of biased ligands for therapeutic use.

## AUTHOR CONTRIBUTIONS

**Ami Nakajima:** Writing – original draft; investigation; validation; visualization; formal analysis. **Hiroto Kaneko:** Investigation; validation; visualization; formal analysis. **Kosuke Oyama:** Investigation; validation; formal analysis; visualization. **Misumi Kuchiji:** Investigation; validation; formal analysis; visualization. **Ayane Itakura:** Investigation; validation; formal analysis; visualization. **Chiaki Arai:** Investigation; validation. **Mitsunori Shiroishi:** Conceptualization; methodology; data curation; investigation; validation; formal analysis; supervision; funding acquisition; visualization; project administration; writing – original draft; writing – review and editing; resources.

## FUNDING INFORMATION

This work was funded in part by JSPS KAKENHI Grant Numbers JP25709080 and JP15K14460 to M.S.; the Platform Project for Supporting in Drug Discovery and Life Science Research (Platform for Drug Discovery, Informatics, and Structural Life Science) from MEXT and AMED, and the Kato Memorial Bioscience Foundation to M.S.

## CONFLICT OF INTEREST STATEMENT

The authors declare no conflicts of interest.

## Supporting information


**APPENDIX S1:** Supporting information.

## Data Availability

The data that support the findings of this study are available from the corresponding author upon reasonable request.
