# Supplementary material for: Activity‐restoring mutations in the histamine H3 receptor increase constitutive activity and reduce structural stability
Source: Protein Sci. 2025 Dec 22;35(1):e70408. doi: 10.1002/pro.70408 (PMC12720417; doi:10.1002/pro.70408)
Supplement: Supplementary file 1 — APPENDIX S1: Supporting information. [file PRO-35-e70408-s001.pdf]

Supplementary materials

Activity-restoring mutations in the histamine H_3 receptor increase constitutive activity and reduce structural stability

Ami Nakajima, Hiroto Kaneko, Kosuke Oyama, Misumi Kuchiji, Ayane Itakura, Chiaki Arai, Mitsunori Shiroishi

Department of Biological Science and Technology, Tokyo University of Science, 6-3-1 Niijuku, Katsushika-ku, Tokyo, 125-8585, JAPAN

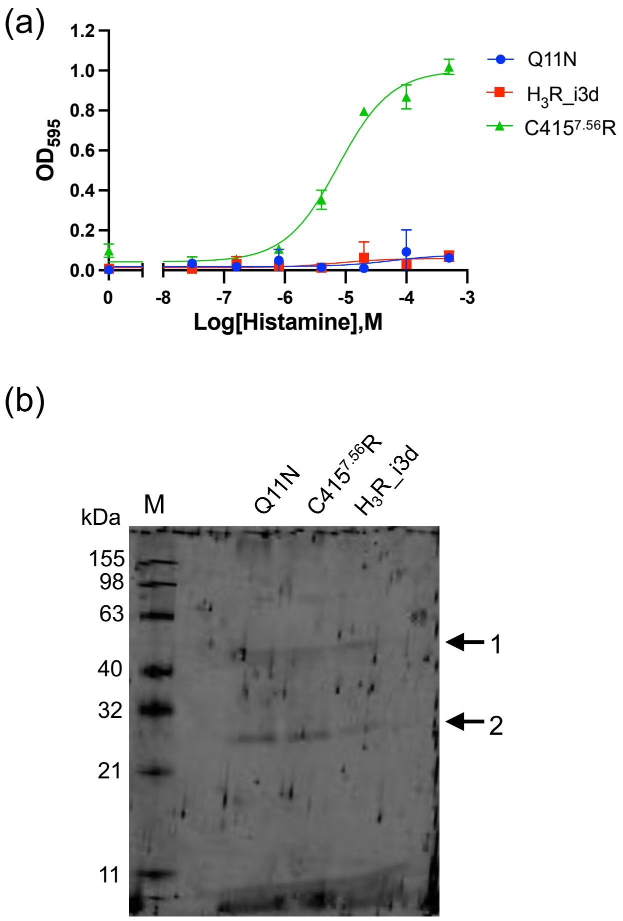

Supplementary Figure 1

(a) Histamine dose–response growth curves of unmutated H_3R_i3d and the Q11N mutant, with the $C415^{7.56}R$ mutant included as a positive control. (b) In-gel fluorescence analysis after SDS-PAGE of unmutated H_3R_i3d and the mutants expressed in YB1 cell. Arrow 1 indicates the band derived from H_3R_i3d-GFP observed between 63 and 40 kDa. Arrow 2 indicates free GFP resulting from degradation of the fusion protein. M, molecular size marker.

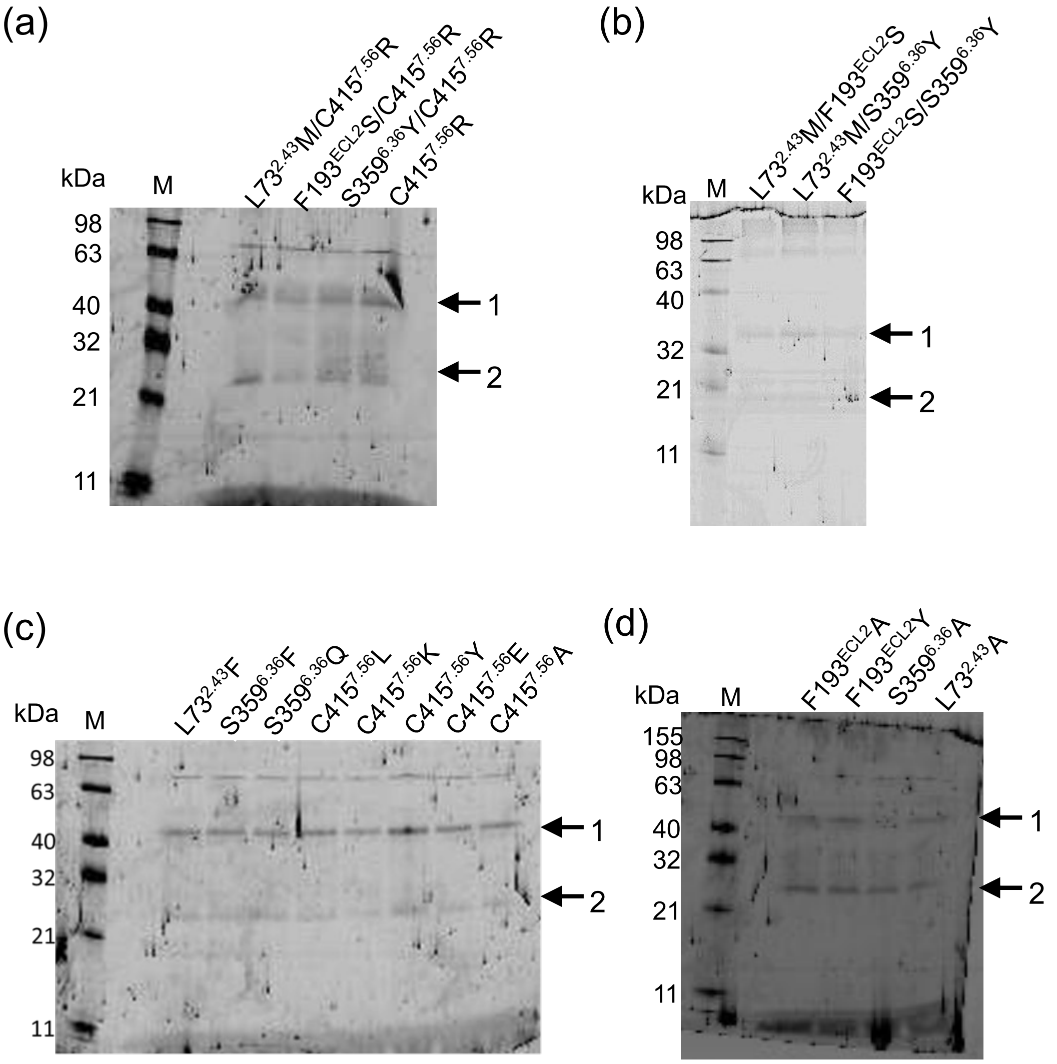

Supplementary Figure 2

(a-d) In-gel fluorescence analysis after SDS-PAGE of the H_3R_i3d mutants expressed in YB1 cell. Arrow 1 indicates the band derived from H_3R_i3d-GFP observed between 63 and 40 kDa. Arrow 2 indicates free GFP resulting from degradation of the fusion protein. M, molecular size marker.

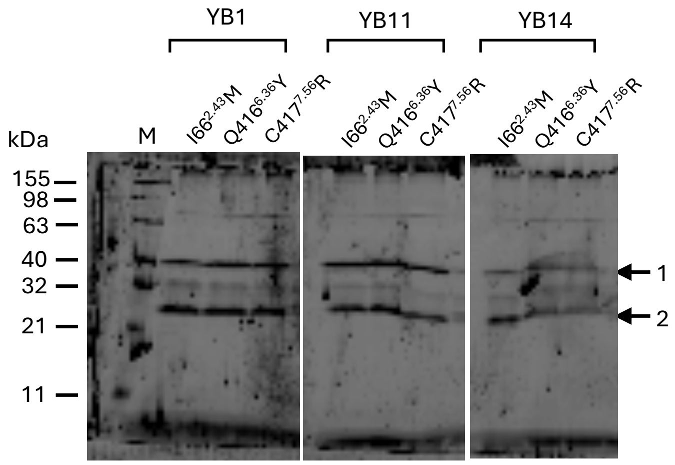

Supplementary Figure 3

In-gel fluorescence analysis after SDS-PAGE of the H_1R_i3d mutants expressed inYB1, YB11, and YB14 cells. Arrow 1 indicates the band derived from H_1R_i3d-GFP observed near 40 kDa. Arrow 2 indicates free GFP resulting from degradation of the fusion protein. M, molecular size marker.

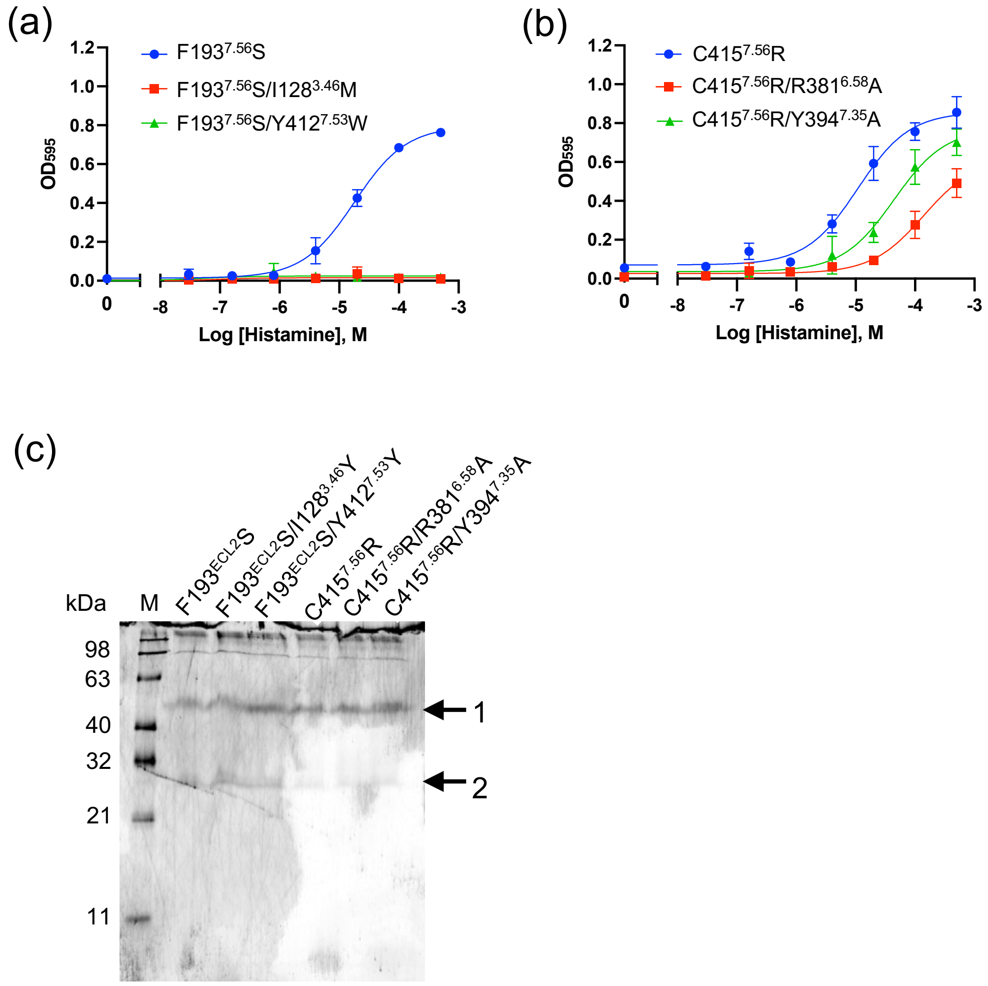

Supplementary Figure 4

(a,b) Histamine dose–response growth curves of H_3R double mutants carrying additional substitutions in residues located near the activity-restoring sites L73$^{2.43}$ and F193^{ECL2}. (a) Double mutants combining substitutions in residues contacting L73$^{2.43}$ (I128$^{3.46}$Y and Y412$^{7.53}$Y) with the F193^{ECL2}S mutation. (b) Double mutants combining substitutions in residues contacting F193^{ECL2} (R381$^{6.58}$A and Y394$^{7.35}$A) with the C415$^{7.56}$R mutation. Experiments were conducted using the YB1 yeast strain. Each data point represents the mean ± SD from three independent experiments, each performed in triplicate. Growth assay data were fitted using a nonlinear regression model (log[agonist] vs. response). (c) In-gel fluorescence analysis after SDS-PAGE of the H_3R_i3d mutants expressed in YB1 cell. Arrow 1 indicates the band derived from H_3R_i3d-GFP observed between 63 and 40 kDa. Arrow 2 indicates free GFP resulting from degradation of the fusion protein. M, molecular size marker.

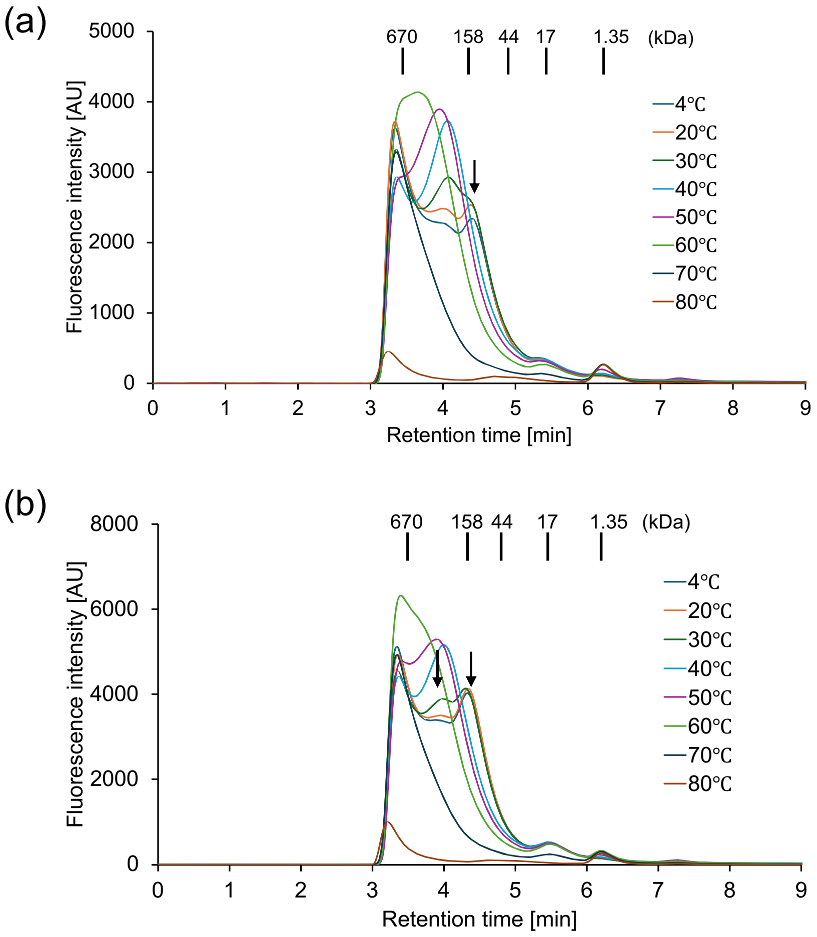

Supplementary Figure 5

FSEC chromatograms of detergent-solubilized unmutated H_3R_i3d(2) (a) and $F193^{ECL2}S$ mutant (b) in 1% DDM/0.2% CHS. After solubilization, samples were incubated for 10 minutes at the indicated temperatures prior to injection. Arrows indicate the monomeric H_3R peak.

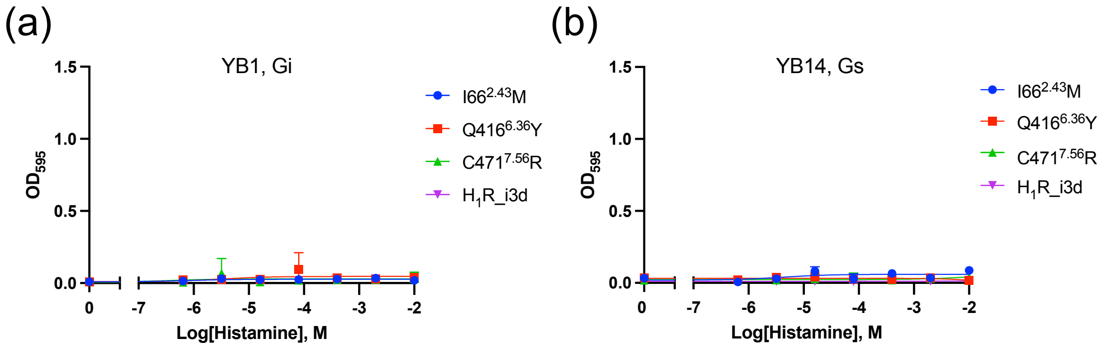

Supplementary Figure 6

Agonist (histamine) concentration–dependent growth curves of unmutated H_1R_i3d and its mutants, obtained using YB1 (a) and YB14 (b) strains.

Supplementary Table 1

Atom–atom contacts (≤ 4.0 Å) within a single H_3 receptor protomer. Entries are restricted to contacts in which the side-chain atom(s) of Leu73$^{2.43}$ (TM2), Phe193^{ECL2} (ECL2), Ser359$^{6.36}$ (TM6), or Cys415$^{7.56}$ (TM7) participate on one side of the pair; the partner atom may be either backbone or side chain. Distances were measured between heavy atoms in the H_3R crystal structure (PDB ID: 7F61), chain A. A label at the end of a row (e.g., "TM7") denotes the transmembrane helix of the partner residue and thus indicates an inter-helical contact. Rows without a TM label correspond to intra-helix (same TM) or intra-loop contacts (for ECL2). Atom names follow PDB conventions; distances are in Å.

			Partner atoms			
	Residue	Atom	Residue	Atom	Distance	
TM2						
	Leu73	CB	Asn70	O	3.24	
			Leu74	N	3.22	
			Phe72	C	3.72	
		CG	Asn69	O	3.57	
		CD1	Asn69	O	3.93	
			His416	CD2	3.77	TM7
			Phe419	CE2	3.73	TM7
			His416	NE2	3.70	TM7
		CD2	Ile128	CD1	3.92	TM3
			Tyr412	CE1	3.82	TM7
ECL2						
	Phe193	CB	Ala190	C	3.94	
			Ala190	O	2.87	
			Phe192	C	3.75	
			Tyr194	N	3.46	
		CG	Arg381	NH1	3.68	TM6
		CD1	Tyr115	OH	3.67	TM3
			Arg381	NH1	3.56	TM6
		CD2	Arg381	NH1	3.64	TM6
			Tyr189	CD2	3.32	
			Tyr189	CE2	3.93	
		CE1	Tyr115	OH	3.86	TM3
			Met378	CE	3.65	TM6
			Arg381	NH1	3.42	TM6
			501(1IB)	CAT	3.99	Ligand
			501(1IB)	FAB	3.45	Ligand
			501(1IB)	CAL	3.78	Ligand
		CE2	Arg381	CZ	3.77	TM6
			Arg381	NH1	3.51	TM6
			Tyr189	CD2	3.72	
			Arg381	NH2	3.81	TM6
			Tyr394	CZ	3.89	TM7
			Tyr394	OH	3.64	TM7
			Tyr189	CE2	3.94	
		CZ	Arg381	CZ	3.80	TM6
			Arg381	NH1	3.38	TM6
			Tyr394	CE1	3.97	TM7
			501(1IB)	FAB	3.90	Ligand
			501(1IB)	CAL	3.77	Ligand
TM6						
	Ser359	CB	Lys355	O	3.62	
			Val356	O	3.43	
			His416	CE1	3.78	TM7
			Leu360	N	3.10	

			Cys415	O	3.83	TM7
			Lys358	C	3.76	
		OG	His416	ND1	3.72	TM7
			Lys355	O	3.87	
			His416	CE1	3.62	TM7
			Cys415	C	3.31	TM7
			Cys415	CA	3.64	TM7
			Cys415	CB	3.20	TM7
TM7						
	Cys415	CB	Ser359	OG	3.20	TM6
			His416	N	3.35	
			Leu411	O	3.29	
			Leu414	C	3.65	
		SG	Leu411	O	3.41	
			Leu414	C	3.99	

Supplementary Table 2

Atom–atom contacts (≤ 4.0 Å) within a single H_1 receptor protomer. Entries are restricted to contacts in which the side-chain atom(s) of Ile66$^{2.43}$ (TM2), Gln416$^{6.36}$ (TM6), or Cys471$^{7.56}$ (TM7) participate on one side of the pair; the partner atom may be either backbone or side chain. Distances were measured between heavy atoms in the H_1R crystal structure (PDB ID: 3RZE). A label at the end of a row (e.g., "TM7") denotes the transmembrane helix of the partner residue and thus indicates an inter-helical contact. Rows without a TM label correspond to intra-helix (same TM). Atom names follow PDB conventions; distances are in Å.

			Partner atoms			
	Residue	Atom	Residue	Atom	Distance	
TM2						
	Ile66	CB	Val67	N	3.40	
			Asn63	O	3.47	
			Tyr65	C	3.74	
		CG1	Asn63	CA	3.91	
			Asn63	OD1	3.66	
			Gly62	O	3.62	
		CG2	Tyr468	CD1	3.87	TM7
			Tyr468	CE1	3.63	TM7
			Tyr468	CE2	3.93	TM7
			Tyr468	CZ	3.66	TM7
		CD1	Leu121	CD2	3.81	TM3
			Ile420	CD1	3.82	TM6
			Leu121	CD1	3.86	TM3
TM6						
	Gln416	CB	Cys471	CB	3.71	TM7
			Lys412	O	4.00	
			Lys415	C	3.77	
			Ala413	O	3.57	
			Leu417	N	3.17	
		CG	Cys471	CB	3.70	TM7
			Leu417	N	3.99	
		OE1	Arg125	NH1	3.07	TM3
		NE2	Asn472	CB	3.47	TM7
TM7						
	Cys471	CB	Leu470	C	3.59	
			Gln416	CB	3.71	TM6
			Gln416	CG	3.70	TM6
			Asn472	N	3.23	
			Lys415	NZ	3.57	TM6
			Gln416	CA	3.84	TM6
		SG	Leu470	C	3.77	
			Ile467	O	3.82	
			Lys415	NZ	3.12	TM6
			Phe419	CB	3.89	TM6
			Phe419	CG	3.69	TM6
			Phe419	CD2	3.52	TM6
